# A Whole‐Head Finite Element Model for Electrical Neuromodulation via Visual Brain‐Machine Interfaces

**DOI:** 10.1002/advs.202511252

**Published:** 2026-07-16

**Authors:** Shengjian Lu, Tonghe Yang, Yuan Geng, Huan Wu, Yangrui Huang, Te Zheng, Haodi Chen, Shurui Huang, Yi Cao, Jian Yang, Wentao Yan, Yikui Zhang, Wencan Wu

**Affiliations:** ^1^ State Key Laboratory of Eye Health Eye Hospital Wenzhou Medical University Wenzhou China; ^2^ Zhejiang Key Laboratory of Key Technologies For Visual Pathway Reconstruction Eye Hospital Wenzhou Medical University Wenzhou China; ^3^ Eye Research Center, Hangzhou Institute of Medicine, Chinese Academy of Sciences, Eye Hospital Wenzhou Medical University Hangzhou China; ^4^ School of Ophthalmology & Optometry The Eye Hospital Wenzhou Medical University Wenzhou China; ^5^ Beijing Engineering Research Center of Mixed Reality and Advanced Display School of Optics and Photonics Beijing Institute of Technology Beijing China

**Keywords:** computational models, electrical neuromodulation, finite element analyses, optic nerve prosthetics, visual brain‐machine interfaces

## Abstract

Brain‐machine interfaces (BMIs) for vision restoration require models that accurately simulate the anatomy and electrical properties of visual pathways. However, current models focus only on isolated structures, such as the retina or brain, and overlook surrounding tissues. Here, we present a comprehensive computational model of the human head that incorporates the entire visual pathway—including the eye, optic nerve, and brain—along with critical neighboring tissues such as the orbit and paranasal sinuses, thereby enabling precise simulations. Validation using human and large‐animal data shows a strong correlation between the simulated and measured electric potentials. Component‐elimination analysis reveals that the optimized comprehensive model outperforms simplified versions. The model demonstrates its utility in multiple applications: (1) comparative analysis of electrical neuromodulation technologies for optic neuropathy, revealing the electric field intensity limitations of noninvasive approaches and the safety concerns of invasive intraorbital approaches; (2) identification of the optimal stimulation site, showing that transnasal stimulation at the optic chiasm outperforms traditional approaches; and (3) in silico design of electrode arrays for optic nerve prostheses, demonstrating theoretical advantages in invasiveness and visual field coverage compared to existing retinal and cortical prosthetics. This validated and versatile computational resource supports the development of neuromodulation strategies and visual BMI technologies.

## Graphical abstract

A multimodal, personalized head model is presented that integrates the entire visual pathway with surrounding tissues to simulate electrical stimulation of the optic nerve. In vivo validation in humans and goats, plus systematic component‐elimination analysis, demonstrates the high accuracy of the comprehensive model. In silico exploration identifies optimal stimulation sites, safety margins, and prosthetic array designs for next‐generation visual BMIs.

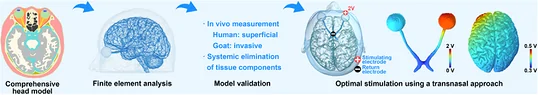

## 1 Introduction

Optic nerve repair represents a critical frontier in visual neuroscience. Optic nerve diseases—including glaucomatous, inflammatory, ischemic, and traumatic optic neuropathies—are the leading cause of irreversible blindness worldwide [1, 2]. Electrical neuromodulation through brain‐machine interfaces (BMIs) has emerged as a promising engineering approach that can either promote guided axonal regeneration [3, 4] or directly elicit visual perception by bypassing damaged retinal circuits [5, 6].

However, the development and optimization of visual BMIs face multiple challenges, among which the lack of comprehensive computational models capable of accurately predicting electric field distributions throughout the entire visual pathway remains a major limitation. Most existing models focus on isolated anatomical structures [7, 8, 9, 10]—such as the retina, optic nerve, or visual cortex. Furthermore, the optic nerve traverses multiple tissue compartments with markedly different electrical properties—from the vitreous‐filled eyeball, through the fat‐rich orbit, the bony optic canal, and adjacent air‐filled paranasal sinuses, to the cerebrospinal fluid‐surrounded brain. Therefore, the lack of a whole‐head, anatomically comprehensive model limits accurate assessment of stimulation efficacy and safety, hindering further technological and clinical advancement.

Here, we developed a comprehensive finite element computational model of the head in both the human and large‐animal model (goat) [11, 12]. This model integrates the entire visual pathway—from the eye to the brain—together with surrounding tissues, including the orbit, paranasal sinuses, skull, cerebrospinal fluid, and muscles. Interestingly, systematic elimination analysis in the computational model showed that omitting individual structures—such as nasal air cavities, orbital fat, cerebrospinal fluid, skull, or muscles—reduced the correlation between simulated and experimentally measured electric fields in the goat model, indicating that these tissues are necessary for achieving accurate model predictions and realistic representations of electric field propagation.

Our model thus enables high‐fidelity simulation of electric field distributions across the visual pathway and its surrounding environment, providing a reliable foundation for the design, optimization, and safe translation of future neuromodulation strategies and visual BMI.

## 2 Results

### 2.1 Personalized Head Model Reconstruction and In Vivo Validation

**FIGURE 1:**
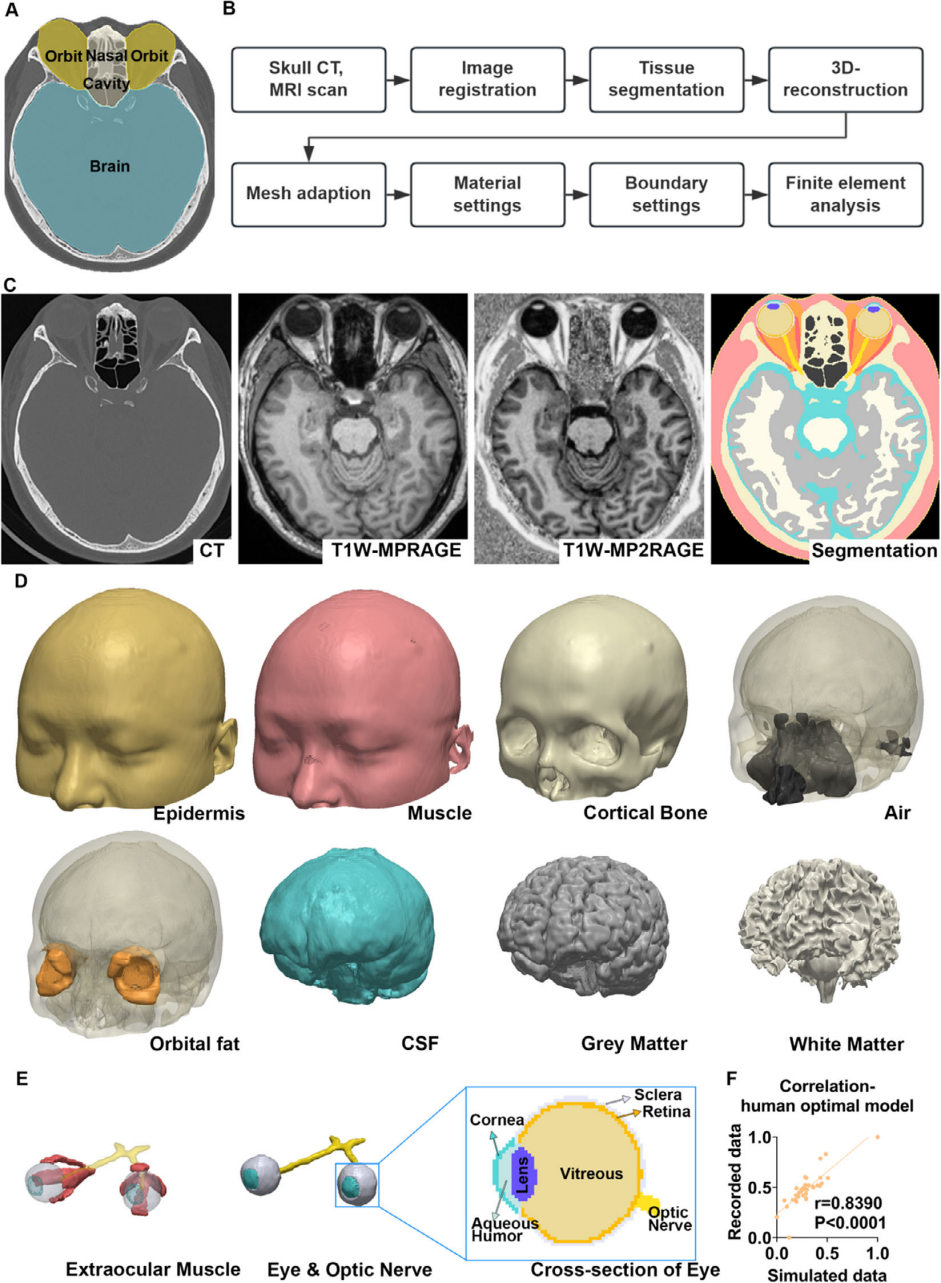
Modeling process of a personalized human head model. A) Overview of the target regions, including orbit, nasal cavity, and brain. B) Flowchart of the modeling and finite element analysis workflow. C) Demonstration of the image registration and tissue segmentation results. D) 3D reconstruction of the anatomical structures, showing individual tissue components such as skin, muscle, cortical bone, air, orbital fat, cerebrospinal fluid (CSF), gray matter, and white matter. E) 3D reconstruction of the extraocular muscles, the eyes (segmented into the cornea, aqueous humor, lens, vitreous humor, retina, and sclera), and the optic nerve. F) Pearson correlation analysis between the normalized recorded and simulated electric potentials for human optimal model (n = 10 human models with 40 sampling sites, r = 0.8390, p < 0.0001, 95% confidence interval: 0.7119 to 0.9128).

Cranial CT and two T1‐weighted MRI sequences [13, 14] from ten adults were registered and segmented (Figure 1A–C). Tissue segmentation delineated air in nasal cavities, cranial bones, intracranial soft tissues (gray and white matter, cerebrospinal fluid), and ocular structures (Figure 1D, E). Synthetic layers representing poorly resolved structures, including a 0.5‐mm skin layer and corneal, retinal, and scleral representations, were added for accurate electrical parameter modeling [15]. A dense mesh was subsequently generated for finite element electrical simulation. These procedures (Movies S1 and S2) generated personalized models of the visual pathway and surrounding structures for electrical simulation.

To validate the accuracy of our computational model, we performed noninvasive electrical stimulation and simultaneous measurement on the scalp. We then simulated identical stimulation parameters in our head model and compared the extracted electric potential values with experimental measurements. Comparative analysis revealed a robust correlation in correlation analysis (r = 0.8390, p < 0.0001) (Figure 1F) between measured cranial potential distributions and simulated results.

**FIGURE 2:**
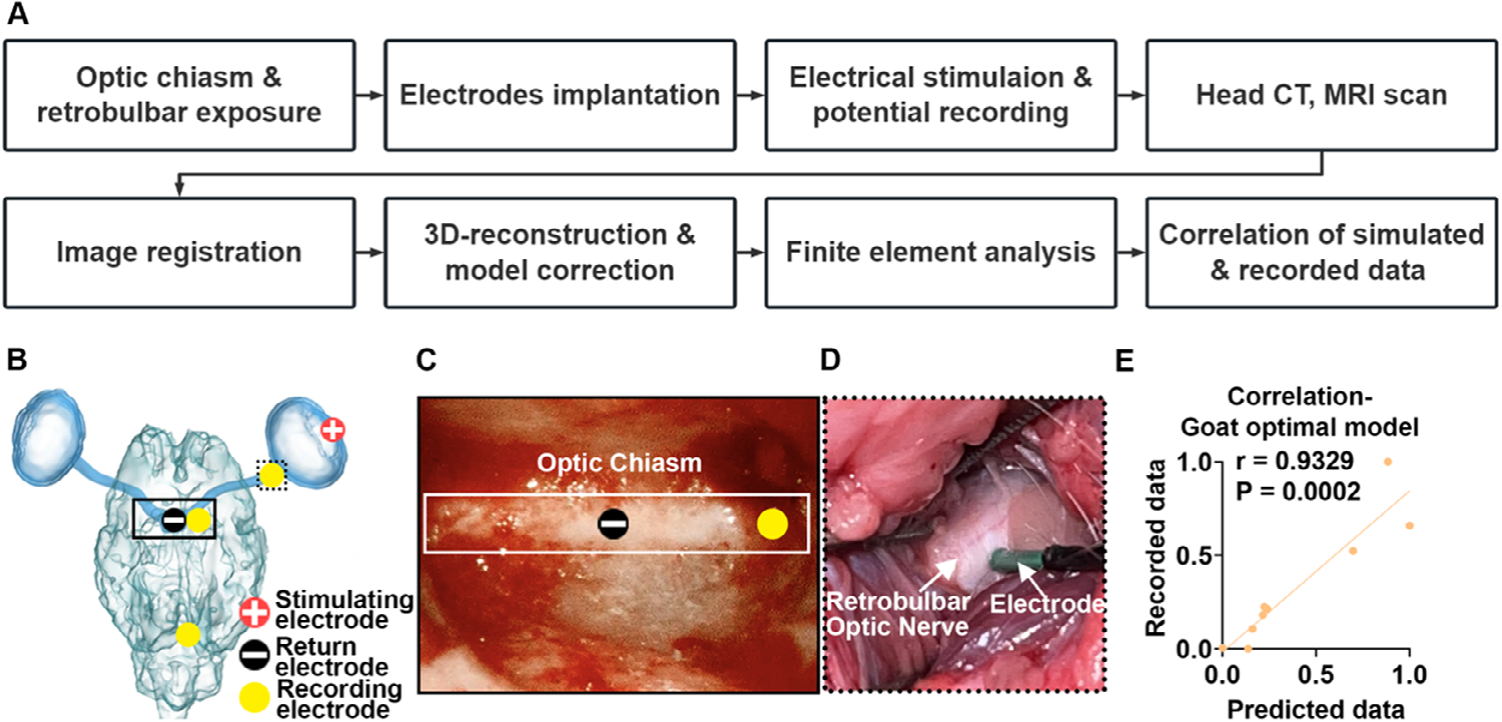
Validation of electric potential distribution and systemic elimination of tissue components on the goat model. A) Flowchart outlining the measurement and finite element analysis processes conducted on the goat. B) Schematic illustration showing the positions of the electrodes in a goat. C, D) Implantation of the recording electrode at the optic chiasm and the retrobulbar optic nerve. E) Correlation between the normalized recorded and simulated electric potentials for goat optimal model (n = 9 sampling sites from 3 goat models, r = 0.9329, p = 0.0002, 95% confidence interval: 0.3214 to 0.9960).

Although human validation demonstrated strong correlations, measurements were limited to superficial recording sites due to ethical constraints. To further validate our model at deeper anatomical sites, we employed a large animal (goat) model, which enabled microinvasive placement of electrodes at the optic nerve [11]. The corneal‐chiasmatic stimulation generated an electric field along the optic nerve, with potentials recorded from electrodes inserted into the optic nerves (Figure 2A–D). CT and MRI were taken after surgery for detailed anatomical reconstruction and precise simulation (Figure S1A‐C). Simulation results demonstrated a gradient potential along the retrobulbar optic nerve toward the optic chiasm as expected (Figure S1D), exhibited a strong correlation (r = 0.9329, p = 0.0002) (Figure 2E), and showed no significant difference (p = 0.8556) (Figure S1E) between in vivo measurements and computational predictions.

### 2.2 Validation of the Head Model Through Systematic Elimination of Tissue Components

To comprehensively assess simulation fidelity, we systematically investigated the impact of tissue component modifications, including the addition of cancellous bone, and elimination of the lens, aqueous humor, air cavities, orbital adipose, CSF, or muscular tissue (Figure S2A–F). Notably, alterations to individual tissue components had minimal effect on simulation accuracy (Figure S2G), likely due to the superficial positioning of recording electrodes, where the electric field is dominated by proximal anatomical structures rather than deeper tissues. Correlation coefficients improved monotonically as stimulation intensity increased.

Systematic elimination of tissue components in the large animal model with deep electrode recordings showed that omitting structures such as lens, aqueous humor, nasal air, orbital fat, CSF, and skull muscle each reduced the correlation coefficient (Figure S3). These results underscore that a comprehensive model including essential surrounding tissues is necessary for reliable electric field simulations. Interestingly, incorporation of cancellous bone slightly reduced simulation accuracy (Figure S3G), consistent with prior research [9, 16].

### 2.3 Non‐Invasive Therapeutic Neuromodulation Technologies: Low Electric Field Intensity along the Optic Nerve

We first used our model to evaluate the efficiency of current noninvasive neuromodulation techniques [17, 18, 19, 20, 21, 22]. Prior work indicates that applied electric fields may promote axonal regeneration [22, 23, 24, 25], but clinical benefits in optic neuropathies have been inconsistent, potentially due to insufficient electric field strength delivered to the optic nerve.

**FIGURE 3:**
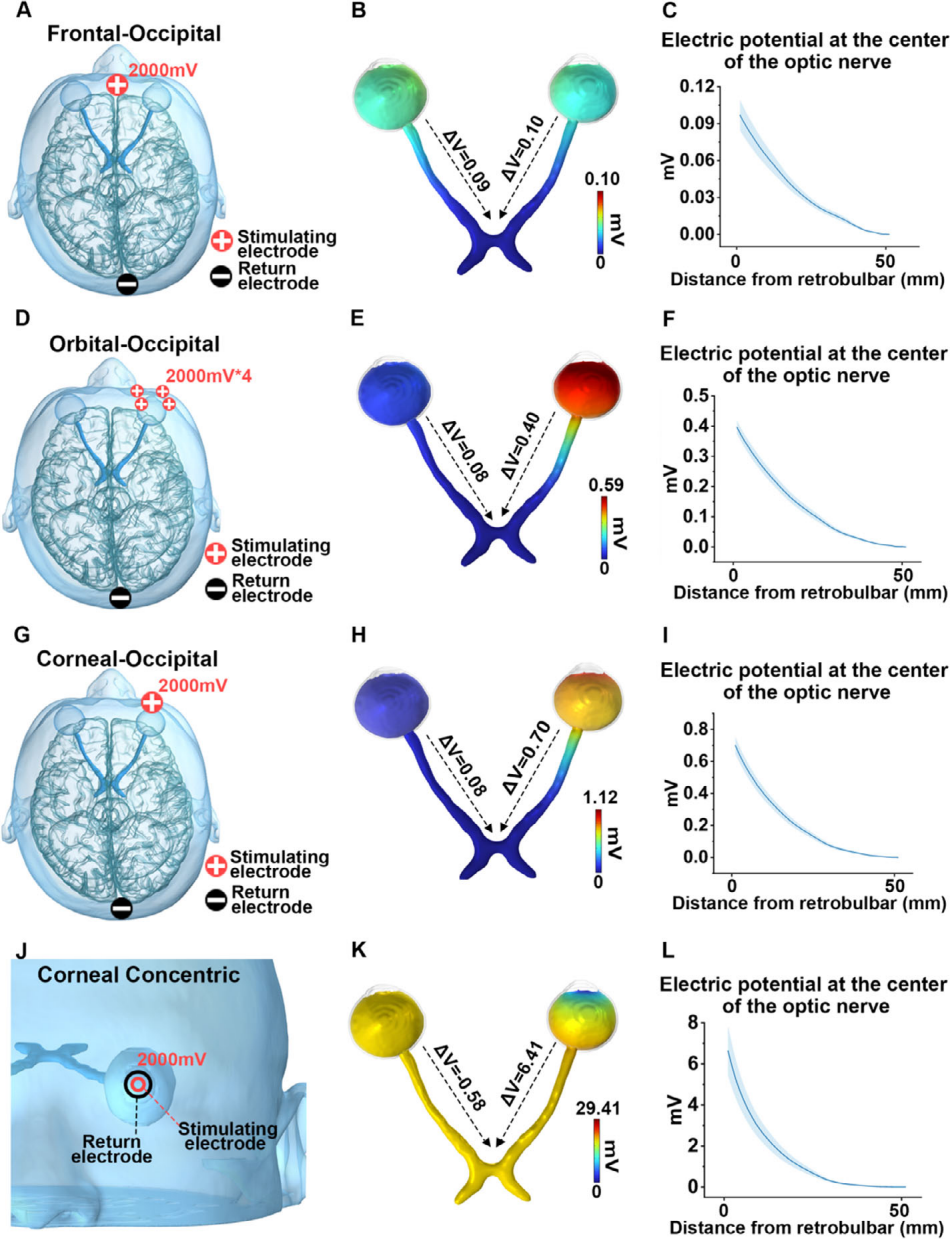
Distribution of electric potential along the optic nerve under noninvasive stimulating approaches. A) Electrode arrangements under the frontal‐occipital protocol. B, C) Illustration and quantification of the electric potential distribution along the optic nerve under the frontal‐occipital protocol. D) Electrode arrangements under the orbital‐occipital protocol. E, F) Illustration and quantification of the electric potential distribution along the optic nerve under the orbital‐occipital protocol. G) Electrode arrangements under the corneal‐occipital protocol. H, I) Illustration and quantification of the electric potential distribution along the optic nerve under the corneal‐occipital protocol. J) Electrode arrangements under the corneal concentric rings protocol. K, L) Illustration and quantification of the electric potential distribution along the optic nerve under the corneal concentric rings protocol. All data are presented as mean ± SEM, n = 10 human models. All units are expressed in millivolts.

Using our validated head model, we analyzed electric field distributions along the optic nerve in ten human subjects under four commonly used noninvasive stimulation protocols. In the frontal‐occipital protocol (Figure 3A), with electrodes positioned along the skull midline, the potential difference across optic nerves was negligible (0.09 and 0.10 mV) (Figure 3B,C). The orbital‐occipital (Figure 3D) and corneal‐occipital (Figure 3G) protocols produced higher ipsilateral potential differences (0.40 mV and 0.70 mV, respectively) (Figure 3E, F, H, I), while maintaining minimal contralateral effects. The corneal concentric ring protocol, in which electrodes were placed directly on the cornea (Figure 3J), generated the largest ipsilateral potential difference (6.41 mV), with a low contralateral effect (−0.58 mV) (Figure 3K, L). All these noninvasive protocols produced electric field intensities across the optic nerve (length: 40 mm) at least 2–3 orders of magnitude below the threshold required for effective directional axonal regeneration as established by previous studies (100 mV mm^−1^) [4].

### 2.4 Invasive Therapeutic Neuromodulation Technologies: Higher Electric Field Intensity with Safety Concerns

**FIGURE 4:**
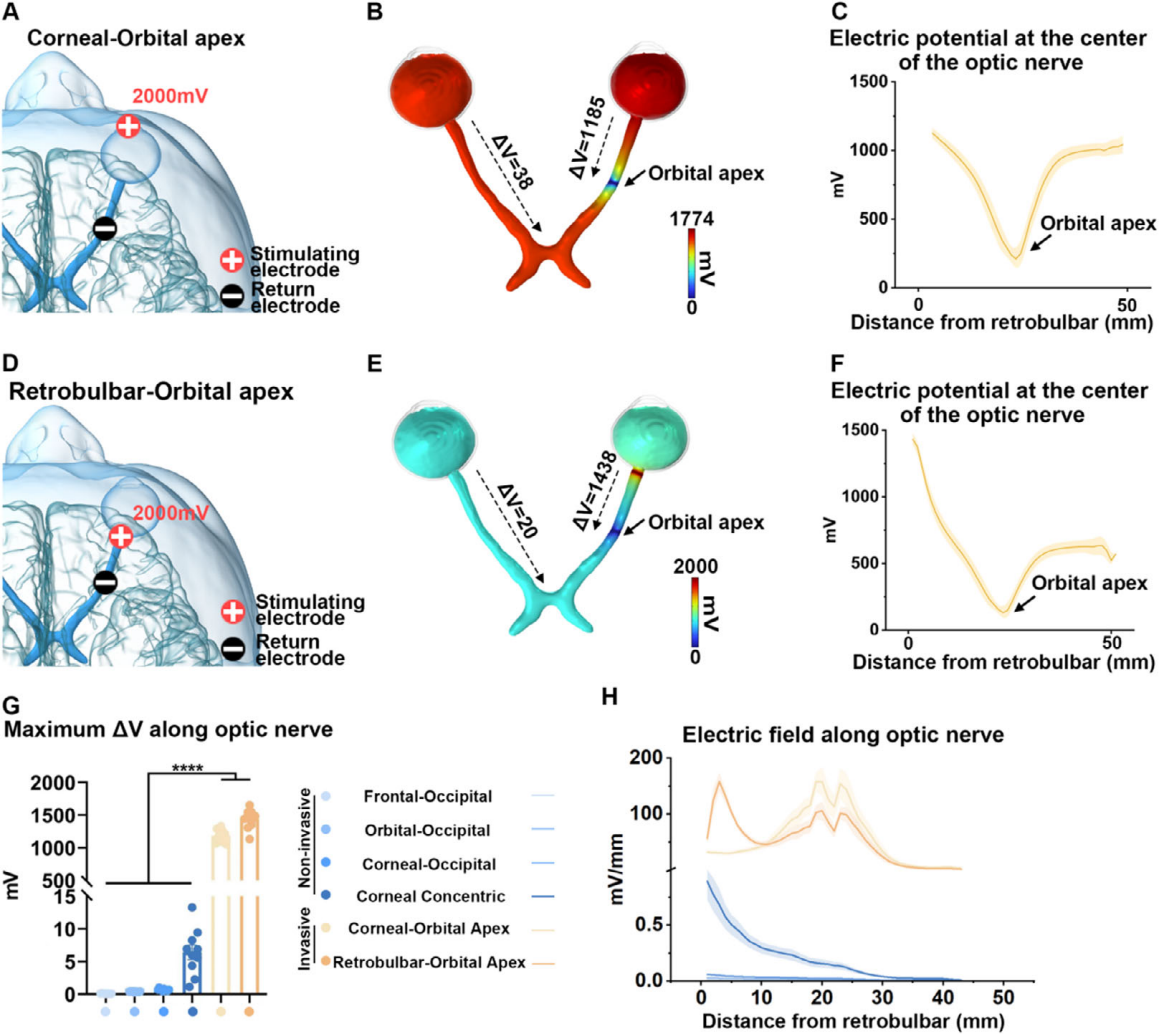
Distribution of electric potential along the optic nerve under intraorbital stimulation approaches. A) Electrode arrangements under the corneal‐orbital apex protocol. B, C) Illustration and quantification of the electric potential distribution along the optic nerve under the corneal‐orbital apex protocol. D) Electrode arrangements under the retrobulbar‐orbital apex protocol. E, F) Illustration and quantification of the electric potential distribution along the optic nerve under the retrobulbar‐orbital apex protocol. G) Comparison of the maximum potential difference (ΔV) along the optic nerve across noninvasive and intraorbital stimulation strategies. Two‐way ANOVA, ^****^: *p* < 0.0001. H) Electric field along optic nerve of noninvasive and intraorbital stimulation strategies. All data are presented as mean ± SEM, n = 10 human models. All units are expressed in millivolts.

**FIGURE 5:**
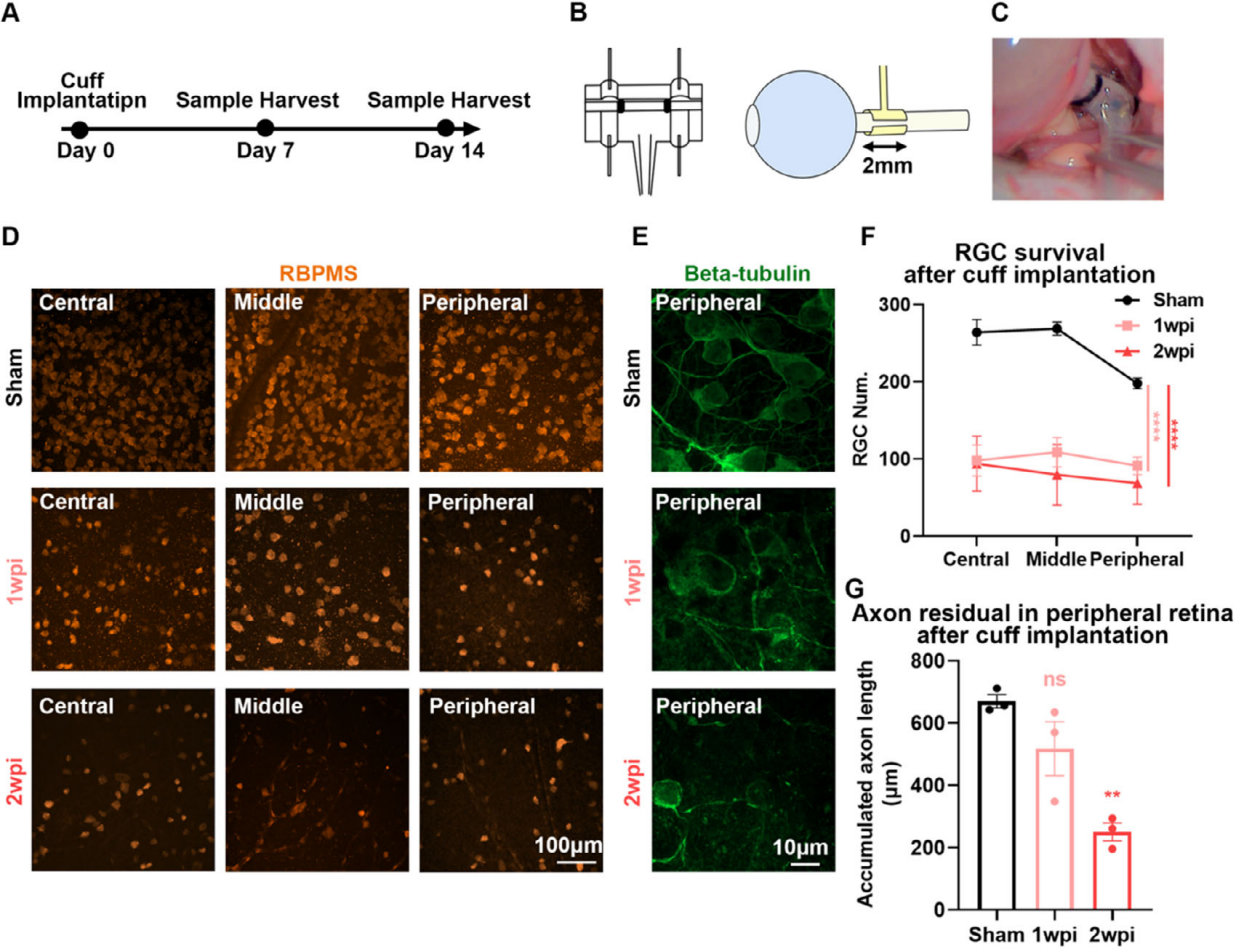
Safety issues of intraorbital stimulation approaches. A) Experimental design for the safety evaluation of the cuff electrode implantation process. B, C) Illustration and microscopic view of the implanted cuff on the retrobulbar optic nerve. D, E) Retinal immunofluorescence staining of RBPMS‐positive RGCs and beta‐tubulin‐positive axons in the sham and cuff‐implanted groups, wpi: week post‐implantation. F) Quantitative analysis of RGCs in all retinal zones following cuff electrode implantation compared with that in the sham group. Two‐way ANOVA, n = 3–4 rats, ^****^: *p* < 0.0001. G) Quantitative analysis of axons following cuff electrode implantation compared with that in the sham group. One‐way ANOVA, n = 3 rats, ns: not significant, ^**^: *p* < 0.01. All data are presented as mean ± SEM.

We next evaluated two intraorbital cuff electrode protocols, which require open‐orbit surgery for placement on the optic nerve [26]. The corneal‐orbital apex protocol (Figure 4A) generated a large potential difference (ΔV = 1185 mV) along the ipsilateral optic nerve (Figure 4B,C), several hundred times higher than non‐invasive methods. The retrobulbar‐orbital apex protocol (Figure 4D) produced an even greater potential difference (ΔV = 1438 mV) and reduced contralateral optic nerve stimulation from 38 mV to 20 mV (Figure 4E,F), indicating greater efficacy and selectivity (Figure 4G,H). However, in rat experiments, intraorbital cuff electrode implantation (Figure 5A–C) led to substantial loss of RBPMS‐positive retinal ganglion cells (RGCs) and beta‐tubulin‐positive axons during two weeks post‐implantation (Figure 5D–G), likely due to mechanical compression from the electrode. The potential safety risks associated with the intraorbital approach suggested that we might need to explore alternative stimulation methods that offer both safety and efficacy.

### 2.5 Identification of Optimal Stimulation Site Using a Transnasal Approach

To address the limitations of conventional methods, we leveraged a transnasal endoscopic approach, a well‐established technique in modern neurosurgery [27, 28]. This approach provides safe and direct access for implantation of the BMI at the optic canal or the anterior optic chiasm. Unlike the orbital segment of the optic nerve, the intracanalicular optic nerve and the optic chiasm do not move during eye movements. Furthermore, the air‐filled sphenoid sinus provides a spacious room for BMI implantation.

**FIGURE 6:**
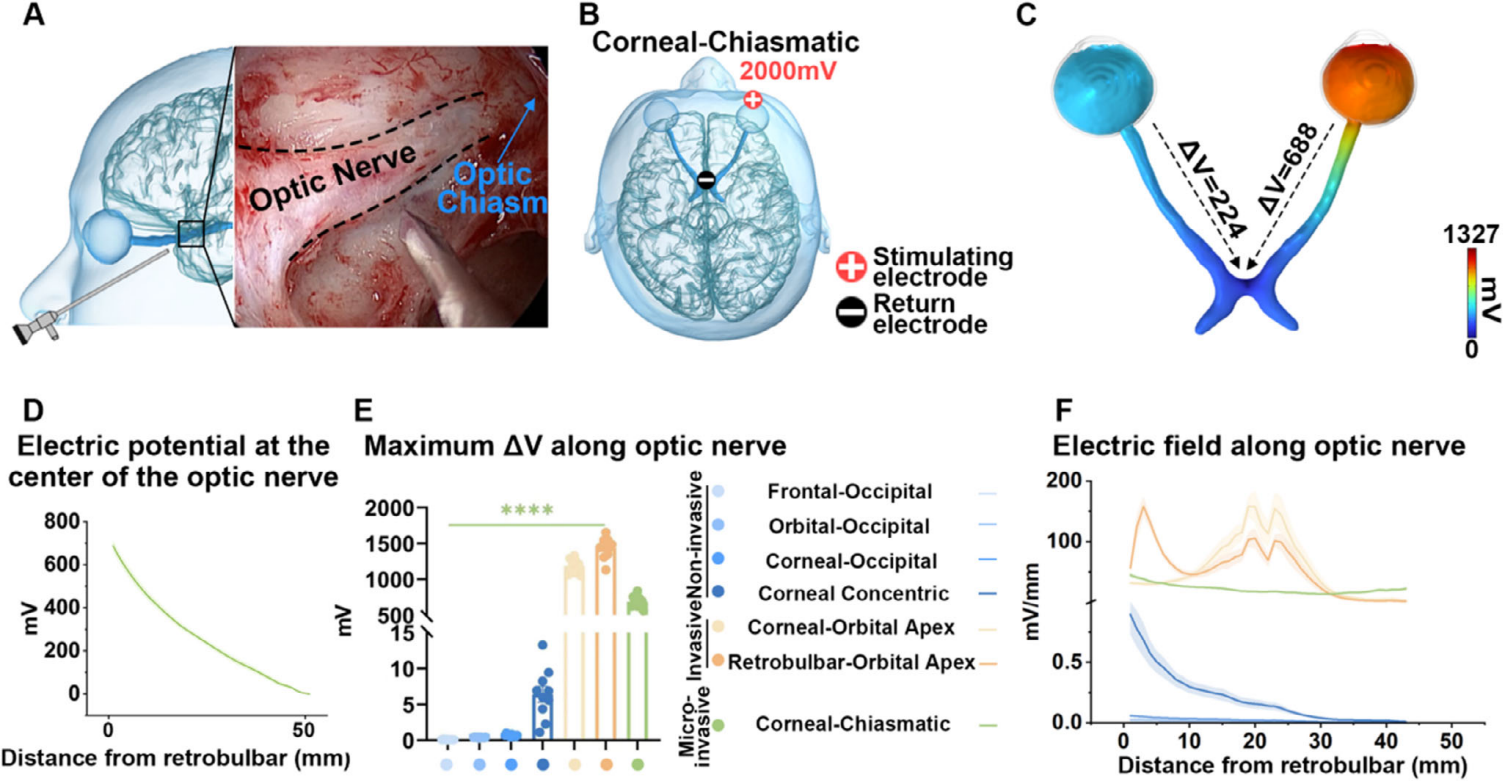
Identification of transnasal electrical stimulation at the optic chiasm outperformed traditional approaches. A) Illustration and endoscopic view of the endoscopic endonasal approach for exposing the optic chiasm. B) Electrode arrangement used in the corneal‐chiasmatic protocol. C) Simulated electric potential distribution along the optic nerve. D) Quantification of the potential distribution along the ipsilateral optic nerve. E) Comparative analysis of the maximum potential difference (ΔV) along the optic nerve across micro‐invasive and other approaches. Two‐tailed paired t test (vs Corneal‐Chiasmatic), ^****^: *p* < 0.0001. F) Electric field along the optic nerve of all stimulation strategies. All data are presented as mean ± SEM, n = 10 human models. All units are expressed in millivolts.

We proposed placing a 180° half‐ring electrode on the anterior optic chiasm during the surgery, paired with a corneal electrode (Figure 6A,B). Simulations showed that this method produced a potential difference comparable (ΔV = 688 mV) (Figure 6C, D) to that achieved with traditional intraorbital electrodes, while delivering a more uniform electric field along the nerve and avoiding the pronounced local perturbations caused by intraorbital implantation (Figure 6E,F). Notably, the corneal‐chiasmatic generated an average electric field across the optic nerve that is 11.5 times greater than that in the brain (Figure S4), enabling effective optic nerve stimulation while maintaining brain safety [29, 30].

### 2.6 Proof‐of‐Concept Designs for Optic Nerve Prosthetic Arrays

Another application of our computational model was to investigate electrode designs for optic nerve BMIs implanted at the nerve surface, offering a potential alternative to retinal or cortical visual prosthetics. A key question is whether a surface‐mounted BMI can activate axons located deeper within the nerve. By varying electrode size, channel count, spatial arrangement, and return electrode position, we found that both the strength and direction of the electric field within the optic nerve could be modulated, suggesting that with appropriate design, any region of the optic nerve could theoretically be activated.

**FIGURE 7:**
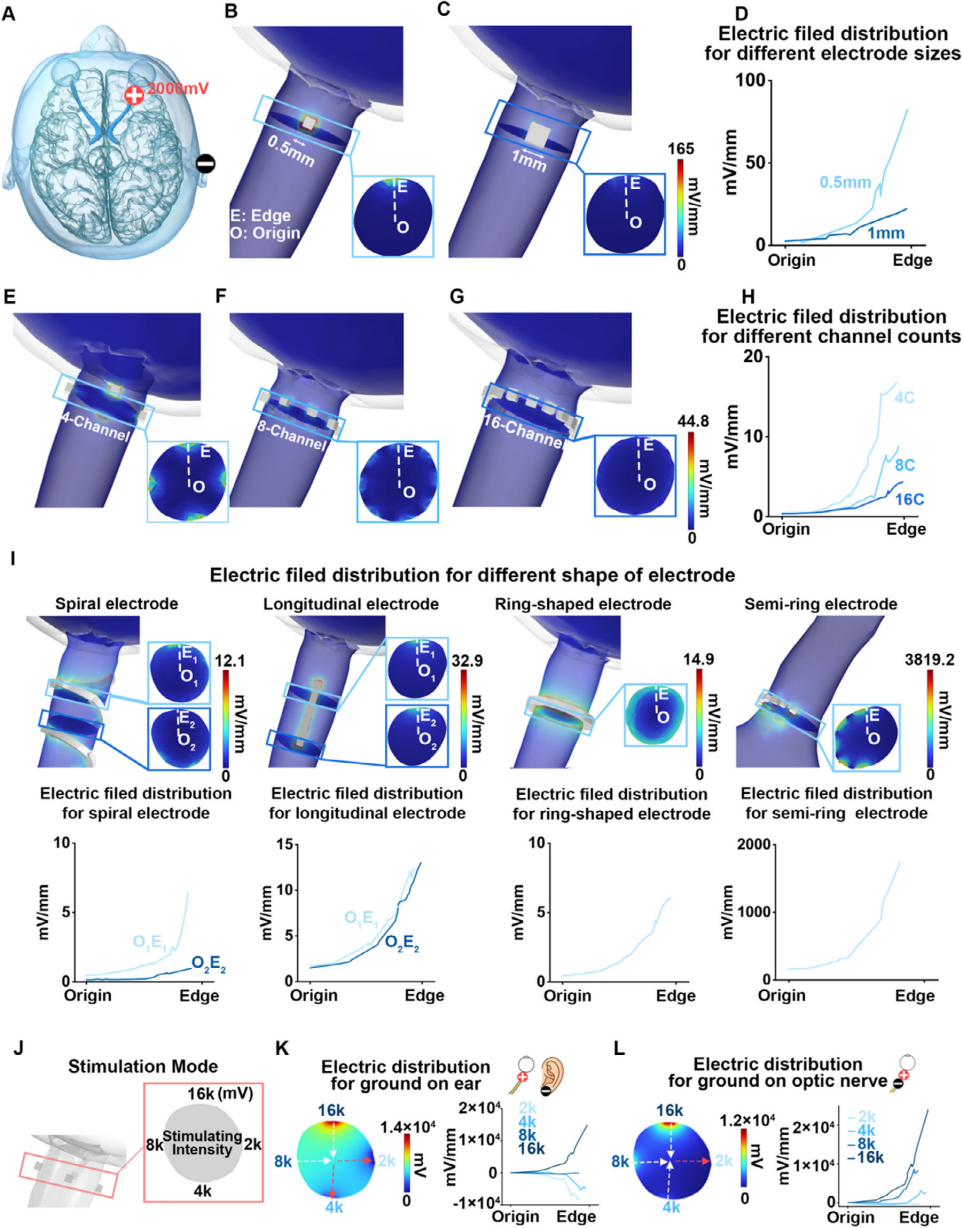
Electric field distributions on the cross‐section across several electrode designs for optic nerve prosthetics. A) Illustration of the position of the optic nerve prosthesis. B, C) Schematics of 0.5 and 1 mm electrode configurations and the cross‐sections of the electrode sites. D) Quantification of the electric field distributions on the cross‐sections generated by electrodes of different sizes. E‐G) Schematics of 4 to 16‐channel electrode configurations and the cross‐sections. H) Quantification of the electric field distributions on the cross‐sections generated by different channel configurations. I) Schematics and quantifications of the electric field distributions on the cross‐section generated by spiral electrodes, longitudinal electrodes, ring‐shaped, and semi‐ring electrodes. J) Illustration of different stimulation intensity among the channels in the 4‐channel setup. K, L) Electric distributions on the cross‐section under stimulation with various intensities (2‐16 V), with the return electrodes placed on the ear and intraorbital optic nerve. The arrows indicated the directions of the electric field. Data from one human model.

Comparisons between 0.5 and 1 mm electrodes placed on the optic nerve surface indicated that smaller electrodes produced more concentrated electric fields with greater intensity along the radial axis, whereas larger electrodes generated broader, but lower‐intensity, field distributions (Figure 7A–D). Increasing the number of active channels from 4 to 16 in electrode arrays reduced the electric field intensity along the radial axis (Figure 7E–H), likely due to a rise in central nerve potential (Figure S5). Exploring different spatial configurations—including spiral, longitudinal, ring‐shaped, and semi‐ring designs—revealed that each arrangement produced distinct internal electric field patterns within the optic nerve (Figure 7I)

Furthermore, varying the voltage input across channels and adjusting the return electrode location allowed control over both the direction and the intensity of electric fields within the optic nerve (Figure 7J). For instance, when the return electrode was on the earlobe, electric fields near the lower voltage electrodes (2 and 4 V) were directed toward these electrodes (Figure 7K), while positioning the return electrode at the intraorbital optic nerve generated electric fields directed away from all electrodes except the 2 V channel (Figure 7L).

## 3 Discussion

In this study, we established the first high‐resolution, subject‐specific computational model integrating the entire visual pathway—from the cornea through the optic nerve, orbit, nasal structures, and brain. By combining CT and MRI imaging, we achieved detailed segmentation of both soft and hard tissues, enabling accurate simulation of electric field distributions throughout these anatomically connected regions. Importantly, the model's predictions were validated by strong correlations with in vivo electrical measurements—both in human patients (superficial recordings) and in large animal models (deep optic nerve recordings)—demonstrating its reliability and relevance for translational applications. Using this validated model, we showcased its utility through three key applications: (1) comparing neuromodulation strategies for optic neuropathy, highlighting limitations of noninvasive and safety issues of invasive methods; (2) identifying transnasal optic chiasm stimulation as an optimal and safer site compared to traditional approaches; and (3) designing and evaluating optic nerve prosthetic arrays. This resource addresses a major gap in previous modeling efforts and offers new insights into designing and optimizing next‐generation neural stimulation strategies and visual prostheses.

### 3.1 Comparing with Previous Computational Models

By leveraging detailed computational modeling and finite element analysis, previous studies have made significant advances in the field; however, these works did not comprehensively include the entire visual pathway along with the surrounding orbit and nasal structures. For instance, Xiaofan Su et al. developed a high‐resolution, full‐eye model—including the cornea, aqueous humor, lens, vitreous body, retina, choroid, and sclera—to simulate retinal electrical stimulation with spatial selectivity. However, their model did not incorporate the optic nerve and brain [7]. Sangjun Lee et al. extended retinal stimulation models by incorporating the skull and brain, allowing for a more comprehensive investigation of how transcranial electrical stimulation affects the retina; however, they excluded the optic nerve and the surrounding orbital and nasal structures [8]. Similarly, Huang et al. constructed anatomically detailed human head models to analyze transcranial electrical stimulation, but did not incorporate the eye, optic nerve, orbit, and nasal cavity [9]. In contrast, our study established a comprehensive model and verified its importance through systematic elimination of tissue components in the large animal model: for example, omitting nasal air or orbital fat in the model reduced the correlation coefficient between the simulated and measured electrical distribution (Figure S2).

Additionally, several previous head models typically relied solely on MRI [31, 32], which provides limited contrast for the skull, CSF, and air cavities—tissues with vastly different conductivities—resulting in challenges for accurate segmentation. By integrating both CT and MRI data, our pipeline achieves precise tissue segmentation, enabling accurate reconstruction of tissue boundaries that are critical for realistic electric field simulations.

### 3.2 Large Animal Model and Cross‐species Translation

The use of a large‐animal model provided key advantages for validating the simulated electric field distributions. Unlike human clinical studies, the large‐animal platform allowed direct measurement of electric fields along the visual pathway through electrode placement within the optic nerve. Moreover, immediate post‐operative CT and MRI imaging were performed to capture anatomical changes following electrode implantation via craniotomy—information essential for constructing anatomically accurate models. In contrast, such real‐time imaging is rarely feasible in human studies. Incorporating these post‐operative anatomical updates markedly improved the reliability of model validation and strengthened the correlation between simulated and experimentally measured electric fields.

While the present study integrates both computational modeling and experimental validation, it is important to acknowledge that human validation remains limited to superficial scalp recordings, which only indirectly reflect electric fields at deeper structures such as the optic nerve and chiasm. Although the large‐animal experiments help bridge this gap by enabling direct in vivo measurements along the visual pathway, they are not intended as direct surrogates for human neuroanatomy. Consequently, caution is warranted when extrapolating these findings to human applications. Future work involving clinical datasets or ex vivo measurements in fresh human cadaver heads will be essential for further validation of deep neural field predictions. Nevertheless, the theoretical framework grounded in quasi‐static Maxwellian volume‐conduction principles, combined with the cross‐species similarity of neural tissue conductivities reported in prior studies [33, 34], lends confidence in the model's generalizability across species.

### 3.3 Optic Nerve BMI: A Promising Pathway Beyond Traditional Visual Prosthetics

Optic nerve BMI represents a promising alternative to current visual prosthetic technologies. The surface areas of the retina (962–1857 mm^2^) [35] and the primary visual cortex (2938‐6296 mm^2^) [36] are substantially larger than the intraorbital optic nerve's cross‐sectional area (3.8–8 mm^2^) [37] in human adults. Consequently, existing retinal or cortical prosthetics face limitations such as restricted visual field coverage and invasiveness associated with implantation via retinal detachment or open‐skull surgery [38, 39, 40, 41]. The larger the visual field coverage desired, the larger—and more invasive—the BMI system typically becomes.

In contrast, an optic nerve BMI could theoretically activate a greater number of axons, enabling a broader visual field with a much smaller implant size [5, 6, 42, 43, 44], potentially implanted via minimally invasive trans‐nasal endoscopy [11]. However, significant technical challenges remain. These include the need for ultra‐high spatial and temporal precision in electric field distribution across the optic nerve to selectively target individual axons. Furthermore, the dura mater and other meningeal layers may impede effective signal transmission, necessitating further research into optimized electrode designs and stimulation parameters. Although the optic nerve's small diameter (∼3 mm, ∼1 million axons) limits current spatial resolution, rapid advances in micro‐electrode fabrication, chip miniaturization, and AI‐based encoding are expected to gradually overcome these constraints [45, 46, 47, 48].

### 3.4 Necessity of Personalized Model

In our study, simulation outcomes differed across head models, and the inter‐individual coefficient of variation varied substantially across electrode configurations (Figure S6), indicating that montage selection is a major contributor to between‐subject variability. Our cohort comprised healthy adults aged 20–40 years; inclusion of participants with different ages would likely further increase inter‐subject variability. Although the small sample size limits generalization, the observed variability patterns provide preliminary insight into the expected range of inter‐subject differences and highlight the importance of individualized modeling. More broadly, because anatomical geometry and tissue conductivity vary widely across individuals, subject‐specific modeling can better inform stimulation protocol design and enhance targeting specificity  [49, 50, 51]. Consistent with this, Huang et al. demonstrated that neglecting personal anatomical features can lead to significant inaccuracies in predicting neural responses [9].

However, the development of a personalized, high‐fidelity computational model remains technically demanding and time‐consuming, requiring approximately 3–5 days per model in our current workflow. Emerging artificial‐intelligence‐based approaches may help automate segmentation and meshing, thereby reducing computation time while improving model accuracy [52, 53].

### 3.5 Limitations

Our study has several limitations. First, the high correlation coefficients are best understood within the context of the limited spatial sampling and their proximity, which was unavoidable due to the small dimensions of the optic nerve. Besides, the anatomical accuracy of the model was constrained by the 1 mm MRI resolution, which required excluding sub‐millimeter structures such as the meninges, vascular networks, and subcutaneous tissue. Without sub‐millimeter imaging of optic nerve microanatomy, the nerve was represented as a uniform cylinder, omitting fine‐scale architecture. This simplification may affect local field estimations: Fiederer et al. reported that large vessels and high vascular density can cause local field errors, whereas vessel‐poor regions are less affected [54]. The dura primarily modulates the overall field amplitude with little effect on its spatial pattern [55]. Manual segmentation also introduced variability in complex regions such as the skull, paranasal sinuses, and CSF spaces. Future studies could improve anatomical fidelity through 3D reconstruction using 7 T MRI or electron microscopy.

Finally, the current electrode array design does not account for the temporal dynamics of electric field changes. Future work should address this to enable the selective activation of optic nerve axon bundles and enhance the performance of visual prosthetics.

## 4 Methods

### 4.1 Study Design

In this study, we aimed to develop a finite element model of the orbital‐nasal‐cerebral region on the basis of skull imaging data obtained from volunteers to investigate the efficacy and safety of different optic nerve stimulation protocols. Ten adult volunteers were recruited, and CT and MRI scans were obtained to construct personalized head models. In vivo measurements of electric potential were taken from volunteers and goats to validate the accuracy of the models. Using these models, we simulated seven electrical stimulation protocols, each characterized by a unique arrangement of electrodes or implantation setups, as well as various electrode designs for optic nerve prosthetics. Experiments on rats (n = 3) were conducted to assess the safety of intraorbital electrodes. Ethical approval was granted by the Ethics Committee of the Eye Hospital of Wenzhou Medical University (Approval No. 2024‐204‐K‐171‐01), and informed consent was obtained from each volunteer. The animal protocols were approved by the Experimental Animal Ethics Committee of Wenzhou Medical University (Approval No. wydw2024‐0241).

### 4.2 Animals

Male Sprague‐Dawley rats were purchased from Beijing Vital River Laboratory Animal Technology Co., Ltd., and housed in the animal facility under temperature‐ and humidity‐controlled conditions with a 12‐h light‒dark cycle. Rodent chow and water were provided ad libitum. All the rats were habituated for at least one week before the experiments commenced, and the rats were aged 2–3 months at the beginning of the study. Male saanen goats aged 4 to 7 months, with body weights of 20–25 kg, were purchased from the Caimu Livestock Company (Hangzhou, China) and housed in the animal facility at Wenzhou Medical University. They were kept in an air‐conditioned room with a normal light/dark cycle and had free access to food and water. The goats also underwent a habituation period of at least one week before the beginning of the experiments.

### 4.3 Image Acquisition and Registration

CT and MR imaging data were acquired to ensure comprehensive anatomical coverage and enable multimodal analysis. CT imaging was performed on a Siemens SOMATOM go. CT scanner (slice thickness = 0.6 mm, X‐ray tube current = 146 mA, Hr60). MRI (Siemens Lumina) data were collected using two T1‐weighted sequences: (1) a 3D magnetization‐prepared rapid gradient echo (MPRAGE) sequence (echo time (TE) = 3.41 ms, repetition time (TR) = 2200 ms, inversion time (TI) = 926 ms, slice thickness = 0.94 mm) and (2) a 3D magnetization‐prepared 2 rapid acquisition gradient echo (MP2RAGE) sequence (TE = 2.89 ms, TR = 5000 ms, slice thickness = 1.14 mm).

Image registration was performed using the FMRIB Software Library (FSL) v6.0.4 to precisely align the CT and MR images. Initially, the CT image was registered to the MR image acquired with the MPRAGE sequence using the FLIRT tool with six degrees of freedom (DOFs) and a mutual information cost function, generating a transformation matrix and an aligned CT image. The MR images acquired with the MPRAGE sequence were subsequently aligned to the MR images acquired with the MP2RAGE sequence using FLIRT with 12 DOF and the same cost function, producing a second transformation matrix. The two matrices were then concatenated using the convert_xfm tool to create a combined transformation matrix, which was applied to the CT image to generate the final aligned CT image in the MRI space of the MP2RAGE sequence. This workflow, which is based on mutual information and affine transformations with trilinear interpolation, minimizes distortion and ensures high anatomical consistency, providing a robust foundation for subsequent analyses such as tissue segmentation, structural quantification, and image fusion.

### 4.4 Tissue Segmentation and 3D Modeling

The registered CT and T1‐weighted image data were imported into 3D Slicer (v5.6.2) for analysis, where manual segmentation was performed on the basis of different grayscale values.

We first delineated key structures from CT images, including low‐signal air (< ‐300 HU) in the nasal cavities and paranasal sinuses, as well as high‐signal cranial bones (> 300 HU). Subsequent segmentation of cranial soft tissues was accomplished using MRI, permitting clear separation of gray (1000‐2500 relative intensity) and white matter (> 2500 relative intensity) with the MP2RAGE sequence. Intracranial interstitial spaces between neural tissues were systematically classified as CSF. Subcutaneous tissue and muscle were not individually distinguished; instead, the two were combined and treated as a single muscle compartment. Due to poor image resolution of skin, a 0.5‐mm synthetic skin layer was added external to the muscle compartment. Ocular structures were then segmented in detail, including the anterior chamber, lens, and vitreous body. Similarly, we added synthetic representations of the poorly resolved structures: the cornea at the anterior surface of the anterior chamber, and the retina and sclera as concentric outer layers around the vitreous body. The optic nerve, extraocular muscles, and orbital adipose tissue were also systematically segmented.

For particularly thin tissues, such as the orbital bony wall, a meticulous manual editing process was performed to correct incomplete boundaries, ensuring geometrically coherent representations of key tissue regions. Small structures, such as blood vessels, the dura mater, the pia mater, and nerve sheaths, were not segmented. Additionally, other cranial nerves were excluded from the segmentation process because they fell outside the primary focus of this study. After segmentation, any minor holes or irregularities were manually corrected (Smoothing Parameters: Kernel size of 0.5–2 mm). All manual segmentation procedures were carried out by an experienced PhD student in medicine.

### 4.5 Electrical Stimulation Protocol and Electrode Modeling

The following seven electrical stimulation protocols were designed.

Noninvasive approaches: (1) frontal‐occipital protocol: the stimulating electrode was positioned at Fpz, with the return electrode at Oz; (2) orbital‐occipital protocol: the stimulating electrodes were evenly arranged around the periorbital region (four in total), with the return electrode at Oz; (3) corneal‐occipital protocol: the stimulating electrode was positioned at cornea, with the return electrode at Oz; (4) corneal concentric rings protocol: an inner ring with a 5 mm diameter served as the stimulating electrode, and an outer ring with a 10 mm diameter served as the return electrode.

Intraorbital invasive approaches: (1) corneal‐orbital apex protocol: the stimulating electrode was positioned at the cornea, with the return electrode at the orbital apex; (2) retrobulbar‐orbital apex protocol: the stimulating electrode was positioned at the retrobulbar optic nerve, with the return electrode at the orbital apex.

Microinvasive approach: corneal‐chiasmatic protocol: the stimulating electrode was positioned at the cornea, with the return electrode at the chiasm.

Noninvasive corneal/skin electrodes were constructed from copper rings with a diameter of 5 mm and a thickness of 0.5 mm. For all noninvasive protocols, conductive paste was applied between the skin and electrodes to ensure good electrical contact. The retrobulbar and orbital apex electrodes were also copper rings, with diameters ranging from 4 to 6 mm, whereas the chiasmatic electrode was a 180° half‐ring. Each electrode was placed in the model and geometrically fitted to the curved surfaces as needed.

### 4.6 Mesh Generation and Finite Element Simulation

The combined model (head and electrodes) was imported into COMSOL Multiphysics 6.2. The mesh generation strategy was optimized to account for the model's anatomical complexity and the available computational resources. Tetrahedral elements were used to discretize the entire domain, with finer local meshes applied in thin or geometrically complex regions (e.g., cornea, retina) to capture high‐gradient fields (Average Mesh Density: 5 million tetrahedral elements per model). Mesh convergence tests were performed to balance accuracy and computational efficiency.

The finite element analysis was executed using the stationary study of the AC/DC module of COMSOL Multiphysics 6.2. Tissue electrical properties (conductivity and relative permittivity) were assigned to brain tissues, the skull, ocular components, and other relevant tissues; the specific tissue electrical property values are provided in Table 1. The initial electric potential of all tissues was set to 0 V. The electrodes were modeled as copper, with a relative permittivity of 1 and conductivity of 5.96 × 10^7^ S/m. All surfaces of the stimulating electrode were set to a potential of 2 V, and all surfaces of the ground electrode were set to 0 V. All external surfaces of the entire model were assigned electrical insulation conditions. The resistance of the reference electrode was set to 50 ohms by default.

**TABLE 1:** Tissue electrical properties.

Tissue	Conductivity [S/m]	Relative permittivity
Air	1 × 10^−14^	1
Skin	2 × 10^−4^	1.14 × 10^3^
Muscle	2 × 10^−1^	2.62 × 10^7^
Cortical bone	2 × 10^−2^	1.05 × 10^5^
Sponge bone	7 × 10^−2^	2 × 10^7^
gray matter	2 × 10^−2^	4.52 × 10^7^
White matter	2 × 10^−2^	3.5 × 10^7^
CSF	2	1.09 × 10^2^
Orbital fat	3.5 × 10^−2^	1 × 10^7^
Optic nerve	6 × 10^−3^	4.01 × 10^7^
Retina	2 × 10^−2^	4.52 × 10^7^
Sclera	5 × 10^−1^	5.1 × 10^6^
Cornea	4 × 10^−1^	4.01 × 10^7^
Aqueous fluid	2	1.09 × 10^2^
Vitreous	1.5	99
Lens	0.2	6.14 × 10^3^

The model enforces charge conservation (current continuity). Under the quasistatic approximation of Maxwell's equations, the following relationships were considered:

(1) ∇·J=Q(l,v)

For passive tissues, *Q(l, v) = 0* to ensure charge conservation.

(2) J=σE+Je

J includes the tissue conduction current (*σE*) and the externally injected current (*Je*).

(3) E=−∇V

The electric field *E* is determined by the gradient of the electric potential *V*. Once *V* is determined, *E* and J can be computed with the model.

The finite element method was applied to numerically resolve these complex electric field equations, utilizing a robust edge element discretization strategy complemented by advanced preconditioning algorithms to efficiently resolve the sparse linear systems. After these equations were solved, 3D distributions of the electric potential *(V)*, electric field *(E)*, and current density *(J)* were obtained. The magnitude and distribution of the electric fields in key regions (e.g., optic nerve, chiasm, and visual cortex) were analyzed, enabling comparisons of the electric fields generated with different electrode placement strategies.

### 4.7 In Vivo Electrical Stimulation and Measurements in Humans and Goats

In the human study, noninvasive electrical stimulation was provided using a ring‐shaped stimulating electrode placed at Fpz and a reference electrode placed at Oz. Direct current stimulation of 1, 2, and 4 V were delivered, with electric potential measurements recorded from four neuroanatomically defined locations: Fz, Pz, T3, and T4.

For the goat study, microinvasive electrical stimulation was provided by positioning a ring‐shaped stimulating electrode on the cornea and a half‐ring reference electrode at the optic chiasm. Recording electrodes were implanted in key locations, including the retrobulbar optic nerve, pre‐chiasmatic optic nerve, and Oz. After delivering 2 V of direct current stimulation, potential values were recorded from these electrodes.

### 4.8 Optic Chiasm Transsphenoidal Endoscopic Exposure in Goats

The procedure followed a previously published protocol [12]. Anesthesia was induced using intravenous propofol (10 mg/kg) and maintained with 3% isoflurane mixed with oxygen and air at a flow rate of 2 L/min (RWD Life Science Co., Ltd., China), which was delivered via a mechanical ventilator. To minimize intraoperative bleeding, hemocoagulase atrox (2 units per goat) was administered. Prior to surgery, the surgical site was prepared with 20 mL of 5% povidone‐iodine solution (Zhejiang Apeloa Inc., China). A double‐T incision was made in the nasal region, and the periosteum was carefully dissected to expose the nasal bone. The nasal bone was then removed to access the anterior bony wall of the sphenoid bone. The middle and posterior olfactory nerve filaments were excised using an endoscopic microdebrider to obtain a clear surgical field. An artificial sphenoid sinus was created to expose the chiasmatic optic nerve within the sphenoid bone. Using an endoscopic microdrill, circular windows were created at the center and on both sides of the optic nerve canal (chiasmatic and bilateral intracanalicular segments) to further expose the optic nerve.

### 4.9 Retrobulbar Optic Nerve Exposure and Electrode Implantation in Goats

The procedure for lateral orbitotomy to expose the retrobulbar optic nerve was adapted from a previously described method [11]. Briefly, following exposure of the optic chiasm, a 5–6 cm continuous incision was made on the lateral side of the orbit. The orbital bone was partially removed after blunt dissection of the periosteum. During the exposure of the optic nerve, surgical sutures were used to retract the extraocular muscles, preventing potential damage. A needle electrode was then implanted into the retrobulbar segment of the optic nerve. The procedure was performed bilaterally to ensure symmetrical exposure and implantation.

### 4.10 Retrobulbar Electrode Implantation in Rats

To expose the retrobulbar optic nerves of the rats, the instruments were sterilized, and the surgical area was prepared. The rats were anesthetized with isoflurane. A 5 mm incision was made over the lateral canthus, and the region was carefully dissected through the subcutaneous tissue to expose the underlying muscles. Blunt dissection was then performed to access the retrobulbar space, isolating the optic nerve from surrounding tissues. A cuff electrode measuring 2 mm in length with an internal diameter of 1 mm (slightly wider than the optic nerve of the rat) and a thickness of 0.3 mm was subsequently implanted on the optic nerve and securely fixed. The lateral rectus muscle was repositioned, and the skin incision was sutured with 6‐0 nylon. The rat was monitored during recovery on a heating pad. Sham‐operated group underwent the same surgical exposure of retrobulbar optic nerve but did not receive a cuff electrode implantation. Both the experimental group and the sham‐operated group of rats were fitted with Elizabethan collars to prevent them from scratching the wounds and electrodes, which could affect the experimental results.

### 4.11 Quantification of RGCs in Rats

The rodents were euthanized with an overdose of isoflurane, and their enucleated eyes were dissected to form posterior segment eyecups, which were then fixed in 4% paraformaldehyde for 2 h at room temperature. The eyecups were subsequently dissected to create retinal flat mounts. The flat‐mounted retinas were blocked for 2 h at room temperature with blocking buffer containing 10% normal donkey serum and 0.3% Triton X‐100 in PBS. The primary antibody (RBPMS, Proteintech, 15187‐1‐AP; Beta‐tubulin, HUABIO, M1305‐2) was diluted in blocking buffer and incubated for 24 h at 4°C on a shaker. After the flat mounts were washed in PBS with 0.3% Triton five times for 15 min each, they were incubated overnight with Alexa Fluor 488‐conjugated anti‐rabbit and Alexa Fluor 594‐conjugated anti‐mouse secondary antibodies. Following additional washes with PBS, the stained retinas were imaged using confocal microscopy (Cell Observer SD, Zeiss, Germany).

### 4.12 Statistical Analysis

Statistical analyses were conducted using GraphPad (9.0) software. Two‐tailed Pearson correlation analysis was performed between the measured and simulated data following normalization. Two‐tailed Two‐Way ANOVA was used to analyze potential differences and RGC data. A two‐tailed paired *t*‐test was used to compare the electric potential differences along the optic nerve across approaches. Statistically significant differences are indicated by asterisks (^*^ *p* < 0.05, ^**^ *p* < 0.01, ^***^ *p* < 0.001, ^****^ *p* < 0.0001). The data are presented as the mean ± SEM.

## Author Contributions

**Shengjian Lu**: Data curation, Formal analysis, Software, Validation, Visualization, Writing – original draft, Writing – review & editing; **Tonghe Yang**: Investigation, Software, Validation, Visualization, Writing – original draft; **Gengyuan**: Investigation, Validation, Writing – review & editing; **Huan Wu**: Investigation, Methodology; **Yangrui Huang**: Investigation, Methodology; **Te Zheng**: Investigation, Methodology; **Haodi Chen**: Visualization; **Shurui Huang**: Writing – review & editing; **Yi Cao**: Writing – review & editing; **Jian Yang**: Writing – review & editing; **Wentao Yan**: Funding acquisition, Writing – review & editing; **Yikui Zhang**: Conceptualization, Data curation, Formal analysis, Funding acquisition, Project administration, Supervision, Writing – original draft, Writing – review & editing; **Wencan Wu**: Funding acquisition, Project administration, Supervision.

## Conflicts of Interest

The authors declare no conflicts of interest.

## Supporting information




**Supporting File 1**: advs76270‐sup‐0001‐SuppMat.docx.

**Supporting File 2**: advs76270‐sup‐0002‐MovieS1.mp4.

**Supporting File 3**: advs76270‐sup‐0003‐MovieS2.mp4

## Data Availability

Requests for information and resources should be directed to the lead contact, Yikui Zhang (zhang.yikui@wmu.edu.cn). The data of the head model is available at https://drive.google.com/drive/folders/1ObU08fK7WEDn7mEaHxoFnUi7Auxszq1D?usp = sharing
