# Supplementary material for: A Whole‐Head Finite Element Model for Electrical Neuromodulation via Visual Brain‐Machine Interfaces
Source: Adv Sci (Weinh). 2026 Jul 16:e11252. Online ahead of print. doi: 10.1002/advs.202511252 (PMC13373889; doi:10.1002/advs.202511252)
Supplement: Supplementary file 1 — Supporting File 1: advs76270‐sup‐0001‐SuppMat.docx. [file ADVS-9999-e11252-s003.docx]

Supporting Information

# **A Whole-Head Finite Element Model for Electrical Neuromodulation via Visual Brain–Machine Interfaces**

***Authors:*** *Shengjian Lu^1, 2#^, Tonghe Yang^1, 2#^, Yuan Geng^1, 2^, Huan Wu^1, 2^, Yangrui Huang^3^, Te Zheng^3^, Haodi Chen^1, 2^, Shurui Huang^1, 2^, Yi Cao^1, 2^, Jian Yang^3, 4*^, Wentao Yan^1, 2*^, Yikui Zhang^1, 2*^ , Wencan Wu^1, 2*^*

**
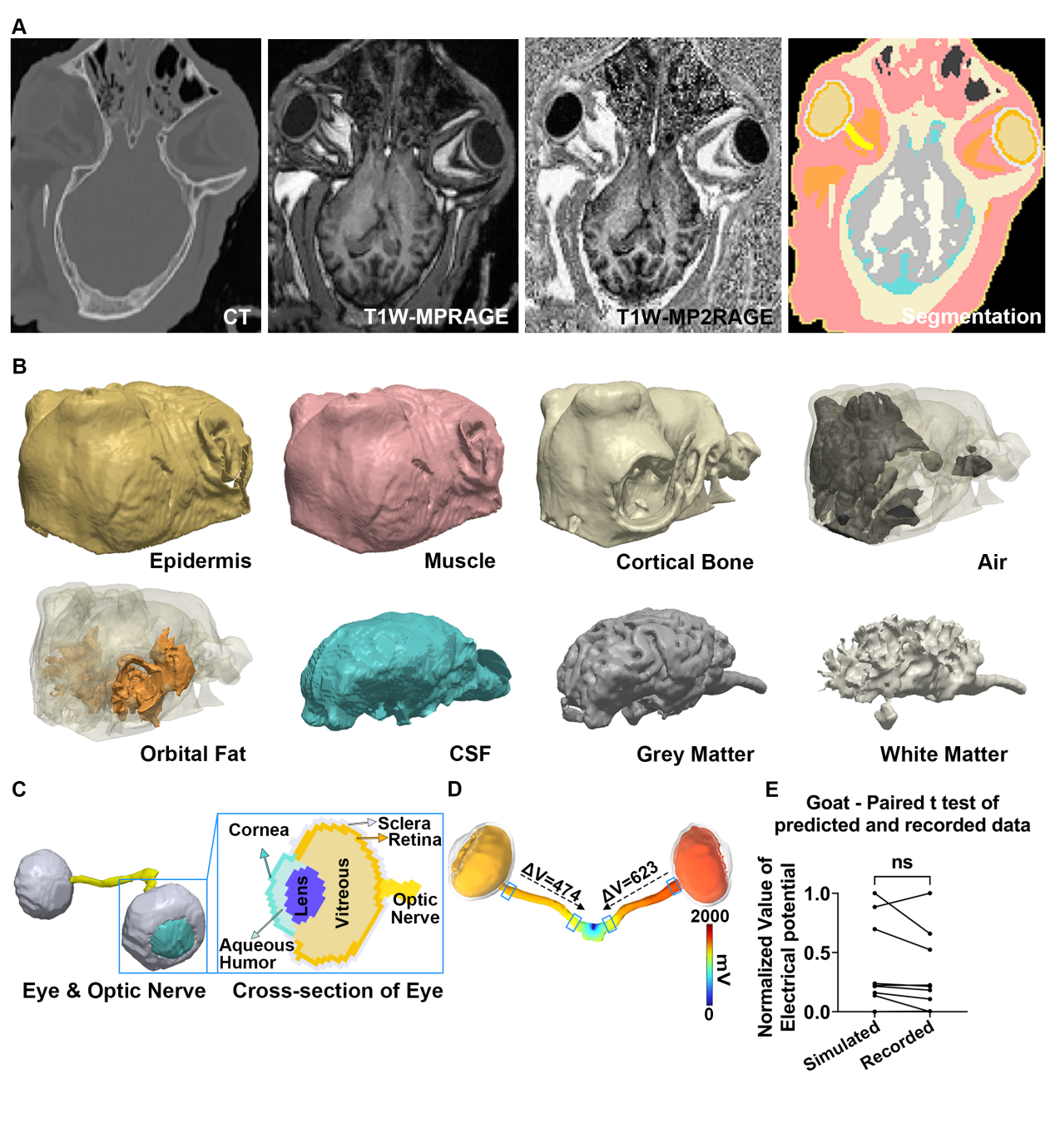
**

**Figure S1**

Modeling process of goat head model. A) Demonstration of the image registration and tissue segmentation results. B) 3D reconstruction of various anatomical structures, including skin, muscle, cortical bone, air cavities, orbital fat, CSF, gray matter and white matter. C) Structural model of the eye and optic nerve, with an enlarged cross-sectional view showing internal components (cornea, lens, aqueous humor, vitreous, retina, sclera, and optic nerve). D) Illustrations of the electric potential distribution in the retina and optic nerve. E) Paired t test comparing normalized recorded and simulated data from 3 goat models, ns: not significant. All units are expressed in millivolts.

**
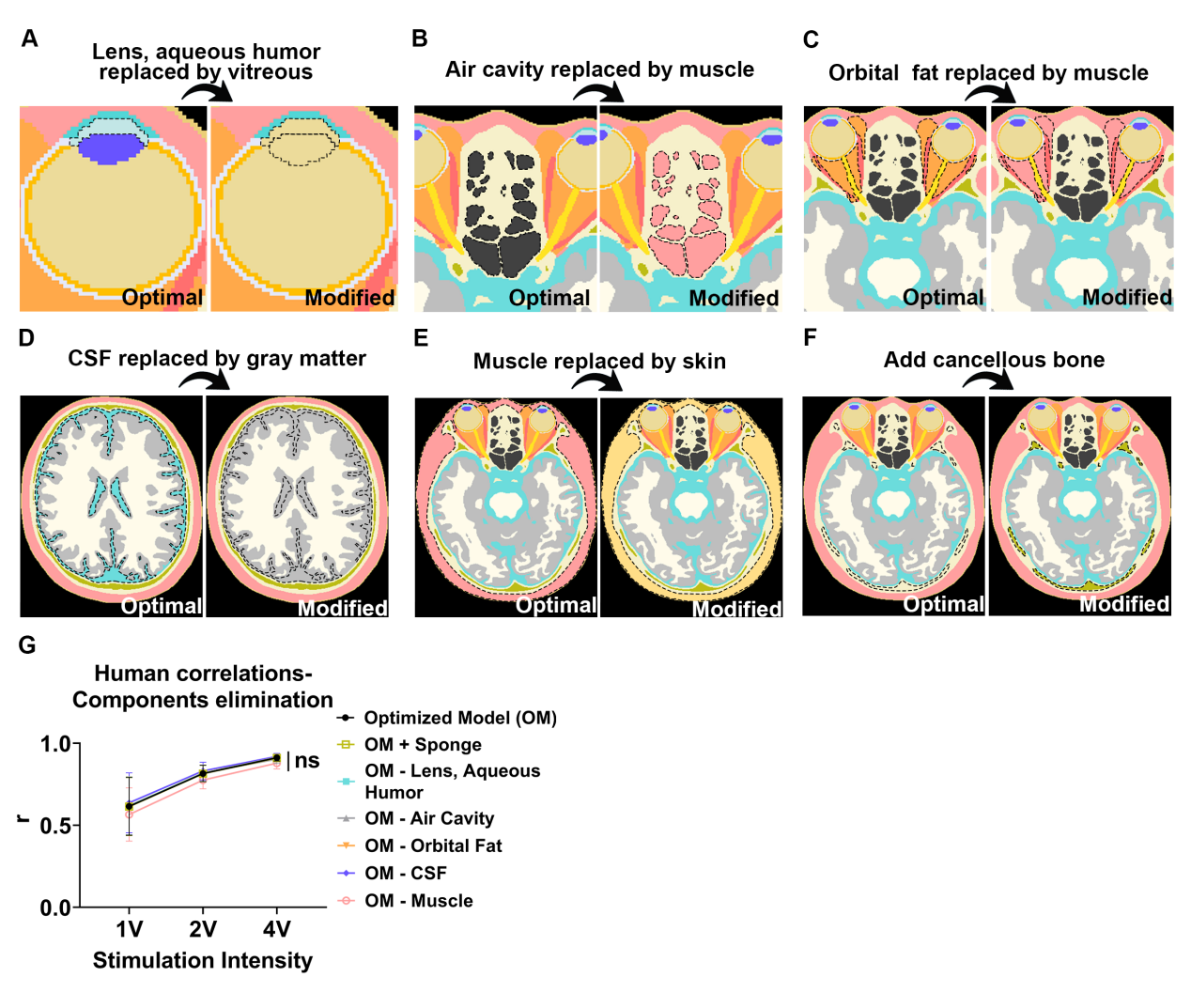
**

**Figure S2**

Impact of component elimination on the accuracy of the human models. A) Illustration of replacing the lens and aqueous humor with vitreous. B) Illustration of replacing the air cavities with muscle. C) Illustration of replacing the orbital fat with muscle. D) Illustration of replacing the CSF with gray matter. E) Illustration of replacing the muscle tissue with skin. F) Illustration of adding cancellous bone. G) Comparison of correlations between simulated and measured data across component-elimination conditions. Two-way ANOVA, data are presented as mean ± SEM, n = 10 human models, ns: not significant.

**
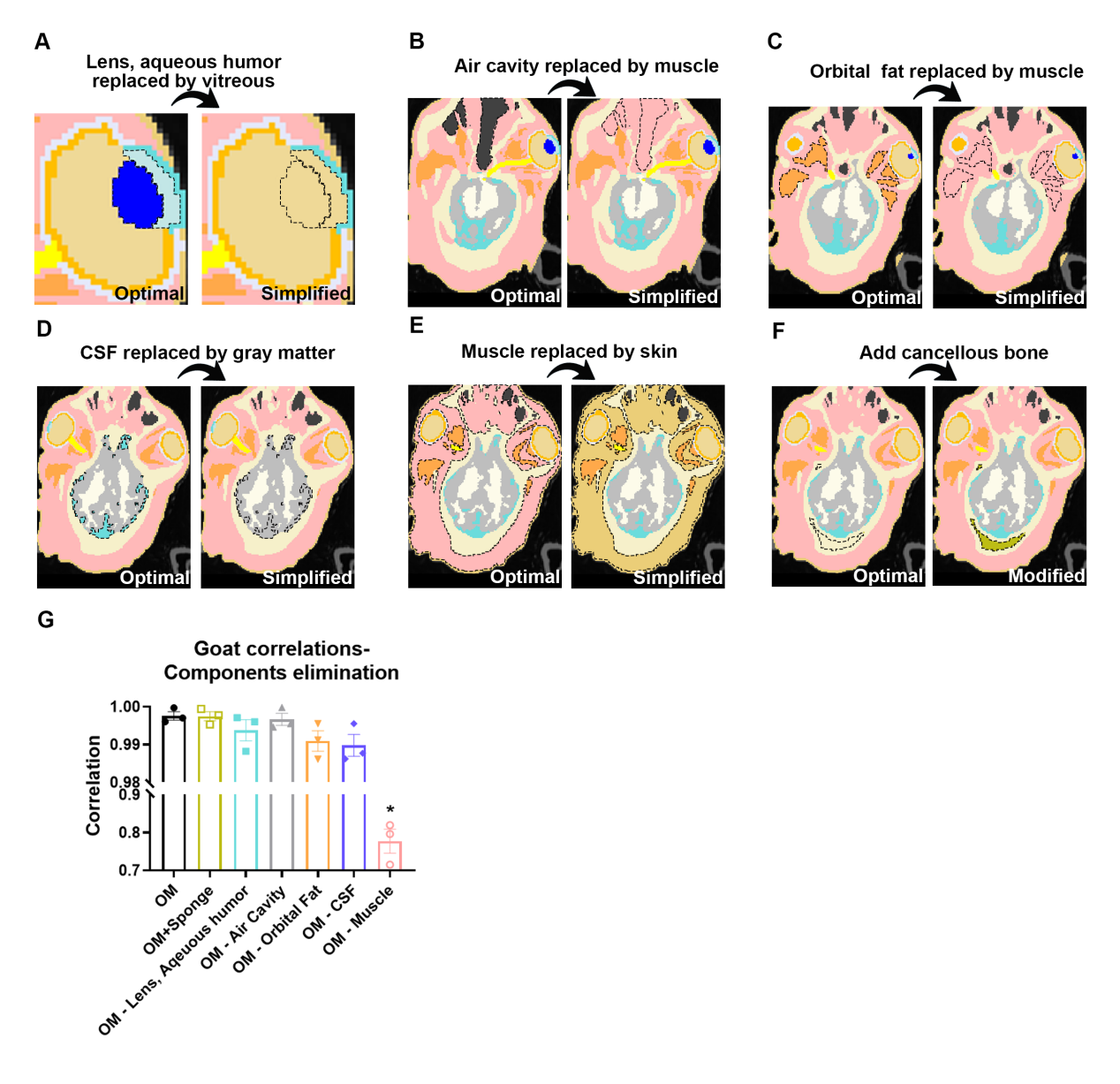
**

**Figure S3**

Impact of component elimination on the accuracy of the goat models. A) Illustration of replacing the lens and aqueous humor with vitreous. B) Illustration of replacing the air cavities with muscle. C) Illustration of replacing the orbital fat with muscle. D) Illustration of replacing the CSF with gray matter. E) Illustration of replacing the muscle tissue with skin. F) Illustration of adding cancellous bone. G) Comparison of simulated-measured correlations across component-elimination conditions. Two-way ANOVA, data are presented as mean ± SEM, n = 3 goat models, ns: not significant, *: P < 0.05.

**
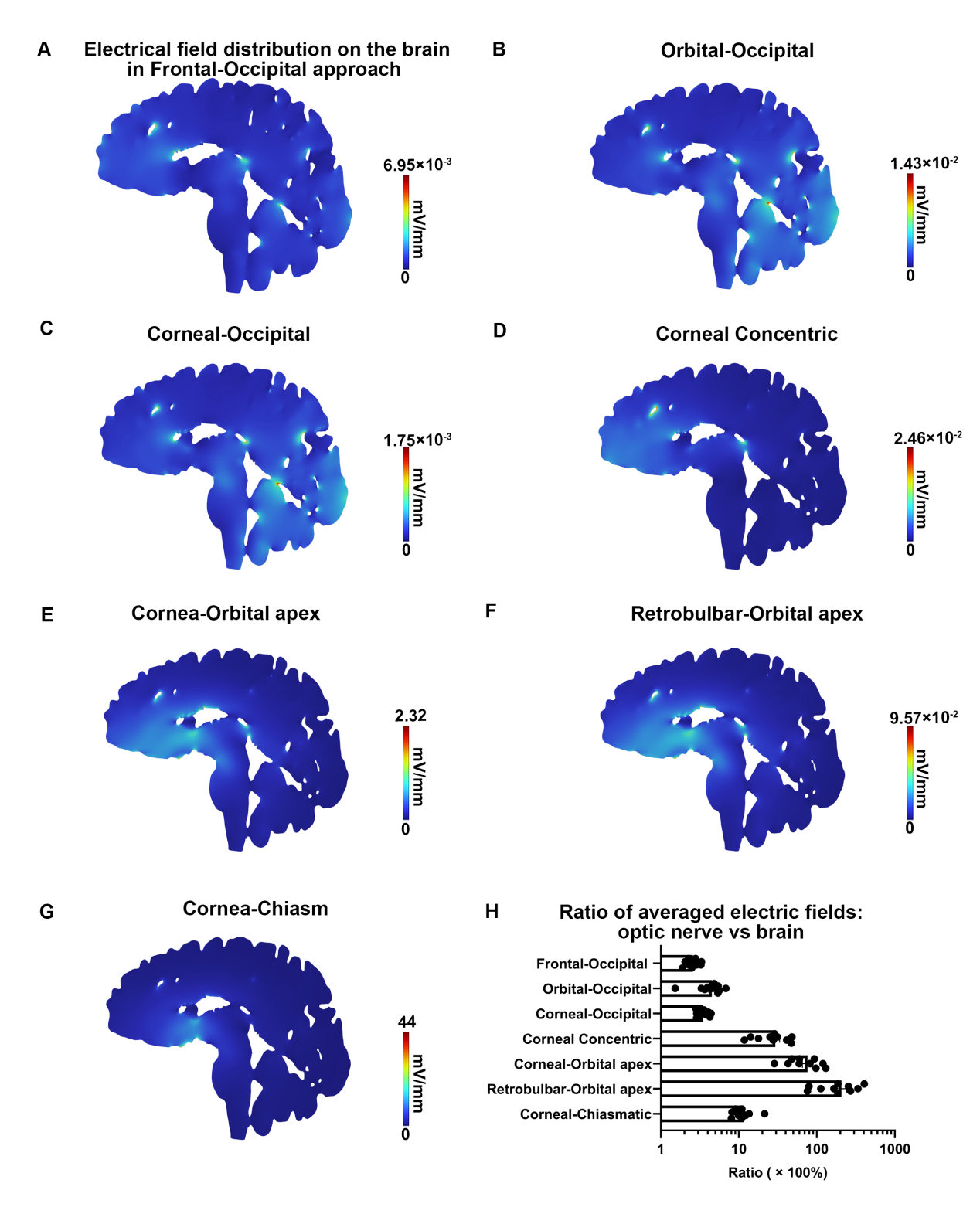
**

**Figure S4**

electric field distribution on the midsagittal plane of the brain across different stimulation approaches with electrodes placed at A) frontal-occipital regions, B) orbital-occipital regions, C) corneal-occipital regions, D) corneal concentric regions, E) corneal-orbital apex regions, F) retrobulbar-orbital apex regions, and G) corneal-chiasmatic regions. H) Ratio of averaged electric field intensity in the optic nerve to that in the entire brain. Data are presented as mean ± SEM, n = 10 human models.

**
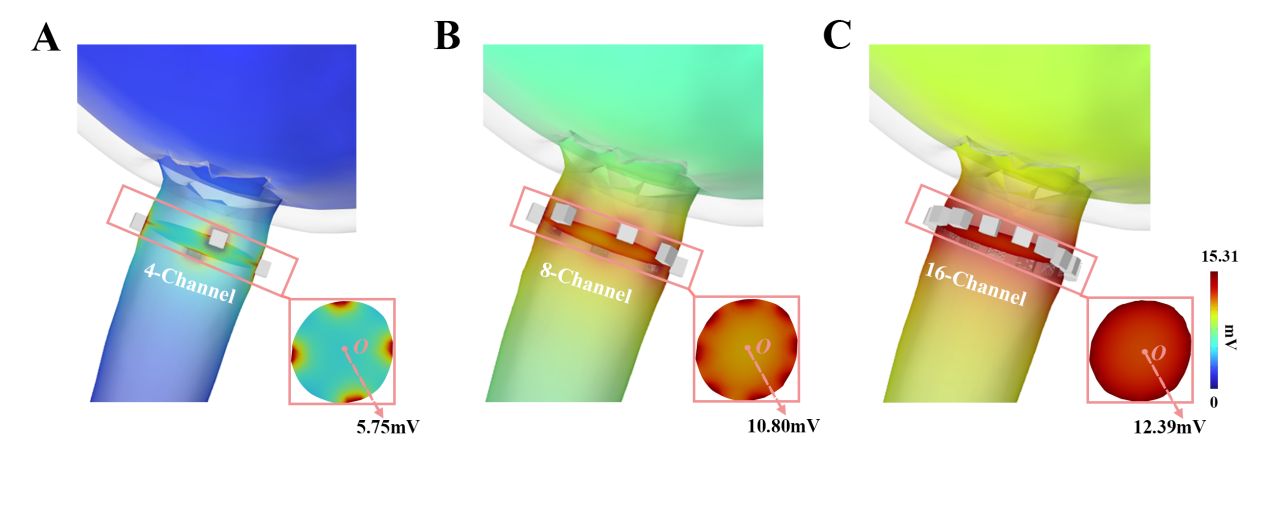
**

**Figure S5**

Electric potential distributions on the cross-section across 4, 8, 16-channel configurations (A-C) for optic nerve prosthetics.

**
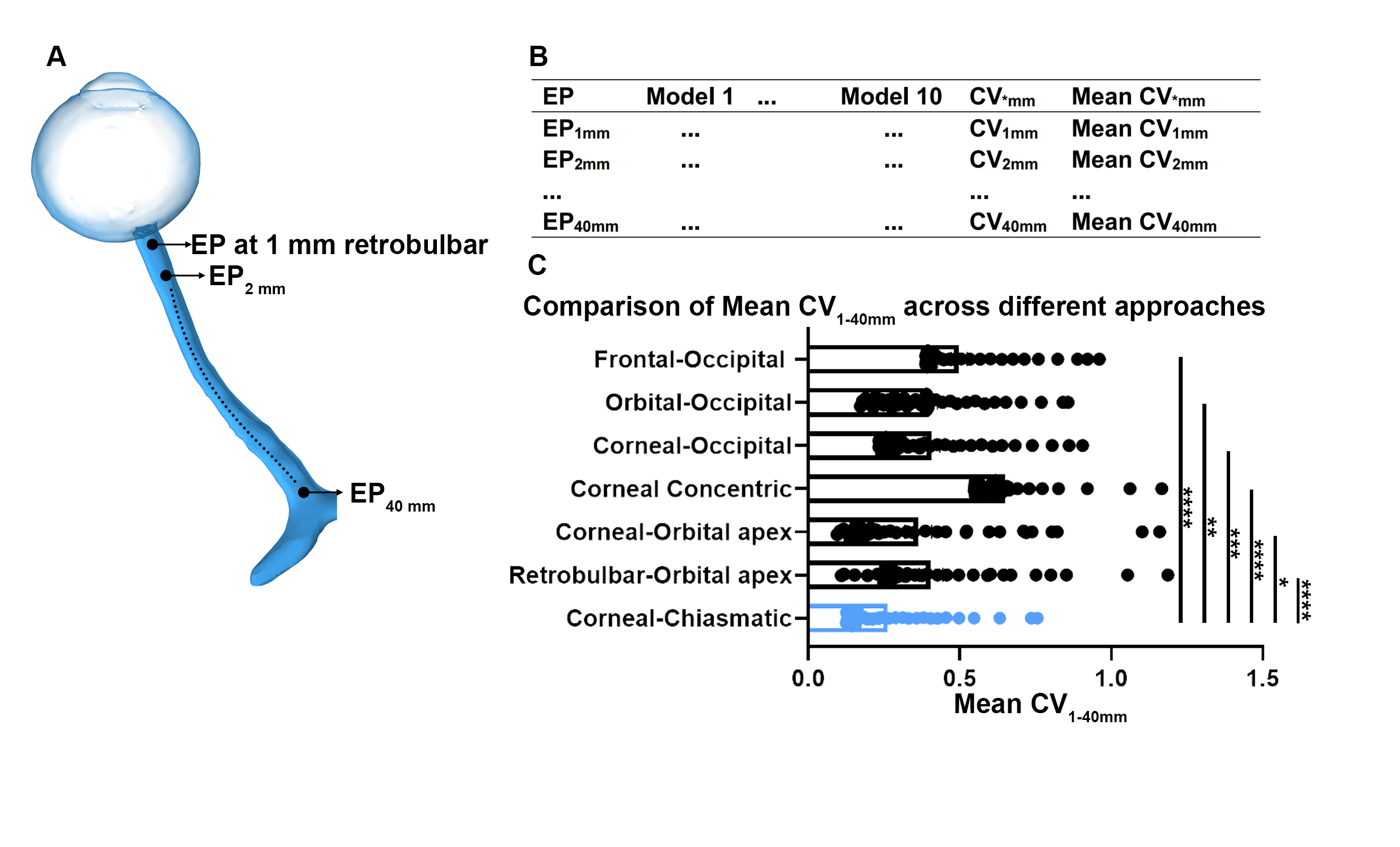
**

**Figure S6**

Variation of electric potential among human models across different approaches. A) Schematic diagram of sampling sites from 1-40 mm behind the optic nerve. B) Demonstration of statistical method to calculate Mean CV_1-40mm_ of 10 human models. C) Comparison of Mean CV_1-40 mm_ across different approaches. One-Way ANOVA, n = 10 human models with 40 sampling sites each, *: p < 0.05, **: p < 0.01, ***: p < 0.001, ****: p < 0.0001. All data are presented as mean ± SEM. EP: Electric Potential. CV: Coefficient of Variation.

Supporting Information

Supplementary movie S1. Step-by-step illustration of the tissue segmentation workflow.

Supplementary movie S2. Step-by-step illustration of the mesh generation workflow from segmented tissue.
